# Does legal freedom satisfy?

**DOI:** 10.1007/s10657-022-09753-6

**Published:** 2022-09-26

**Authors:** Niclas Berggren, Christian Bjørnskov

**Affiliations:** 1 Research Institute of Industrial Economics (IFN), Box 55665, 102 15 Stockholm, Sweden; 2 Department of Economics (KEKE NF), Prague University of Economics and Business, Winston Churchill Square 4, 130 67 Prague 3, Czechia; 3 Department of Economics and Business Economics, Aarhus University, Fuglesangs Allé 4, 8210 Aarhus V, Denmark

**Keywords:** Freedom, Satisfaction, Well-being, Happiness, Civil rights, Rule of law, K10, K38, P10

## Abstract

Much political conflict in the world revolves around the issue of how much freedom to accord people. Liberal democracies are characterized by, e.g., the rule of law and a strong protection of civil rights, giving individuals a great deal of legally guaranteed freedom to lead their lives as they see fit. However, it is not known whether legal freedom suffices to make people satisfied with freedom. Our study explores that issue by relating seven indicators of legal freedom to the satisfaction people express with their freedom of choice. Using a sample of 133 countries over the period 2008–2018, and taking a panel-data approach, we find no robust baseline relationship. However, when exploring conditional associations by interacting the indicators with social trust, the rule of law is positively and increasingly related to satisfaction with freedom above and below a threshold level. Freedom of assembly is more positive for satisfaction with freedom the higher the GDP per capita and in democracies. Thus, for some types of legal freedom, formal legal institutions are complementary with culture, income and the political system in generating satisfaction with freedom.

## Introduction

How much freedom to accord people is one of the most contentious political issues. While liberal democracies provide legal guarantees of a great deal of freedom, not least through the rule of law and civil rights, other forms of government, especially authoritarian ones, are less prone to offer it.^1^ One way to try to justify this kind of legally provided freedom—in arguments for its continued protection in liberal democracies and for its implementation in other forms of government—is to consider its ability to make people satisfied with the freedom to choose what to do in life.^2^ While it may be unwise to opt for “happiness maximization” as a political goal, for reasons outlined by Frey and Stutzer (2000, 2012), it is still conceivable that the legal rules of society can be devised such that people are able to fulfill most of their ambitions in life and become satisfied. This is in fact shown to be the case in over 100 studies relating political, economic and legal institutions to life satisfaction (see Berggren & Bjørnskov, 2020, for an overview).^3^

On that basis, we study how a set of indicators of legal freedom relate to the extent to which people in different countries are satisfied with freedom when it comes to choosing what to do with their lives. The research question is: Does legal freedom suffice to generate such satisfaction, or is something else needed (such as a certain culture, a certain level of national income or a certain political system)? The seven indicators of legal freedom are part of the Human Freedom Index (Vásquez & McMahon, 2020) and capture the rule of law; security and safety; freedom of movement; freedom of religion; freedom of association, assembly and civil society; freedom of expression and information; and freedom of identity and relationships.^4^ The outcome variable is the share of people in different countries who reply that they are satisfied when asked the question “Are you satisfied or dissatisfied with your freedom to choose what you do with your life?” (Gallup, 2020).^5^

We explore this in a panel-data analysis of up to 133 countries over the period 2008–2018. Our baseline results show that few indicators of legal freedom are related to satisfaction with freedom in a statistically significant way, and those that are turn out not to be robustly related to the outcome variable, when taking outliers and variations in the model specification into account. However, when performing interaction analysis with social trust, we find that the rule of law positively affects satisfaction with freedom, and the more strongly the more social trust there is, above a threshold.^6^ When interacting our indicators of legal freedom with national income, the relationship between freedom of association, assembly and civil society and satisfaction with freedom is stronger the higher the income, again above a threshold. Lastly, an interaction analysis with democracy reveals that freedom of association, assembly and civil society generates higher satisfaction with freedom in democracies than in non-democracies.

In other words, for two indicators of legal freedom to really provide satisfaction with freedom of choice, social trust, national income and democracy help. This implies a *complementarity* between formal institutions and culture, positive freedom and political freedom in generating satisfaction with freedom.

This research is inspired by Frey’s (2020, p. 9) assessment of the happiness literature, in which “… much is still unknown, for instance, the importance on happiness of the rule of law, of basic human rights or of types of bureaucracy.” Taking that statement seriously, the contributions of this study are: to bring legal freedom to the forefront of the empirical analysis, including a decomposed study of seven different indicators; to use a new outcome variable, satisfaction with freedom (which is particularly apt for analyzing whether formal institutions encapsulating freedom are able to generate satisfaction with the freedom they create); to undertake an interaction analysis showing under which further conditions legal freedom might generate satisfaction with freedom; and to provide an up-to-date analysis, with data for recent years, covering a large number of countries.

## Literature and theoretical framework

### Literature

While we contribute to the overall literature on satisfaction, we believe our study adds new knowledge, based on our reading of the existing literature, which we summarize briefly here.^7^ An early study, Diener et al. (1995), finds that civil rights are positively related to both life satisfaction and happiness (especially the former) up until the early 1990s, a result that holds for Eastern Europe after the fall of Communism (Hayo, 2007) but that is found by Altindag and Xu (2017) to hold for rich countries only. Veenhoven (2000), however, does not find a statistically significant relationship between either political or personal freedom and happiness for the 1990s, and neither do Ye et al. (2015). Similarly, Welsch (2003) reports that the point estimate for civil rights becomes insignificant whenever income is included in the regressions, suggesting an indirect effect via income. A methodologically different study by Windsteiger et al. (2020) uses the Covid-19 pandemic and ensuing curtailment of civil rights as an exogenous event and study, through a questionnaire, how the intensity by which individuals value freedom of choice affects the consequence of the curtailment for life satisfaction—showing that the stronger one values civil rights, the more life satisfaction was reduced.

There are other studies focusing on freedom. Inglehart et al. (2008) report, for a cross-country sample, a strong positive relationship between the extent to which people felt they have free choice and control over their lives, on the one hand, and subjective well-being (an index of both life satisfaction and happiness), on the other hand. Minkov (2009) confirms the main findings, as does Verme (2009). He shows, using individual-level data, that a measure of freedom of choice and the locus of control predicts life satisfaction better than any other tested factor (such as income, health, marriage, employment and religion). Pitlik and Rode (2016), as well as Nikolaev and Bennett (2016), in turn investigate macro-determinants of individual life control and identify economic freedom as a main factor. Brulé and Veenhoven (2014) find a positive relationship between freedom in the personal sphere and both life satisfaction and happiness (but an even stronger such relationship between psychological freedom—a lack of inner restrictions for seizing opportunities to choose—and those outcome variables). Okulicz-Kozaryn (2015) looks at Eastern Europe and reports that a personal feeling of freedom increases life satisfaction (and more so than elsewhere, and more so than national income). Lastly, there are some studies involving freedom of identity and relationships. Berggren et al., (2017, 2018) for example find that equal rights regarding marriage etc. are beneficial for the quality of life of gay men, as well as for general life satisfaction.

Against this background, our main contribution to the literature is to use a rarely studied but important outcome variable, satisfaction with freedom of choice, and to investigate the institutional, economic and cultural conditions that are conducive to it. To our knowledge, we are the first to conduct such an analysis. Studying satisfaction with freedom of choice rather than more general satisfaction measures, such as life satisfaction, brings more detailed knowledge about people’s evaluations of a key feature of liberal democracy and the market economy. Are people satisfied with freedom of choice specifically? If so, under what conditions?

### Theoretical framework

Our starting point is *legal freedom*, which is the freedom accorded to people according to the legal rules. It is thus a type of negative freedom—the legal rules create a sphere within which people may not be constrained, either by the government, other organizations or other citizens, in executing choices, as long as they do not violate the negative freedom of others (Carter, 2019). Legal freedom consists of two parts: the rule of law and substantive or specified rights. The first part—the rule of law—is the legal system as such, comprising “a number of principles of a formal and procedural character, addressing the way in which a community is governed. The formal principles concern the generality, clarity, publicity, stability and prospectivity of the norms that govern a society” (Waldron, 2020). The second part—substantive rights—specify a number of rights that prohibit or limit interference and discrimination, e.g., freedom of movement; freedom of religion; freedom of association and assembly; freedom of expression; freedom of relationships; and freedom from regulation.

If there is a legal system with a functioning enforcement system abiding by the principles of the rule of law, and if legal rules specify that certain types of behavior are to be allowed or not be constrained, this gives rise to a certain amount of legally protected freedom of choice. People then experience either *satisfaction or dissatisfaction* with that amount of freedom of choice. If they are satisfied, this implies that they do not wish more freedom of choice; if they are dissatisfied, this implies that they do want more freedom of choice.^8^

What speaks in favor of legal freedom having a positive effect on how satisfied people are with the amount of freedom they have? A high amount implies that people have assurance that they can make choices in their lives without being constrained either by public officials or other people. They are thus allowed to try to reach whatever goals they have in life, and if such an ambition, when allowed “free rein” through legal freedom, results in the actual, successful reaching of these goals, or in an expectation that the goals will be reached in the future, satisfaction ensues. Thus, our satisfaction measure is not the same as life satisfaction, but arguably, if the amount of freedom of choice they have allow them to make choices that result in (expected) preference satisfaction, they will tend to be satisfied with the amount of freedom of choice they have. Otherwise, they will be dissatisfied with that amount of freedom and want more of it.^9^ In addition, people may derive “procedural utility” from having legal freedom (Frey et al., 2004).

However, under other circumstances, legal freedom can be unrelated or negatively related to satisfaction with freedom of choice. First, even with freedom people may not reach the goals they want to reach and therefore feel unsatisfied with freedom because they feel dissatisfied with its perceived consequence. To reach goals one not only needs negative freedom but also positive freedom in the form of resources and abilities. Without the latter, frustration and dissatisfaction with the former (since they are insufficient for the reaching of the goals) can ensue. Furthermore, dissatisfaction with the freedom of choice at hand can also stem from the behavior of others. It may be that other people use their freedom in ways that create conflict in society. In trying to reach *their* goals, they may say and do things that are unpleasant to others. Second, if people underestimate the actual freedom of choice in place, then even if they reach their goals, they may not feel satisfied with freedom of choice for that reason. They do not see the clear link from legal freedom to what they have been able to do. Third, if people overestimate the actual freedom of choice in place, and if many of their goals are not reached, they may (erroneously) “blame” freedom of choice, when in actual fact, there was insufficient legal freedom to begin with. In such a setting, it is also probable that some people feel overburdened by the potential for choice accorded them by legal freedom.^10^

This reasoning suggests that it is theoretically ambiguous how legal freedom affects satisfaction with freedom of choice—whether there is an effect and what sign it takes. That is not the end of the story, however. So far, we have discussed the direct relationship between legal freedom and satisfaction with freedom. We furthermore consider the possibility of a cultural factor—*social trust*—influencing this relationship. Our hypothesis is that social trust, in addition to strengthening satisfaction with freedom as such (cf. footnote 4), interacts with legal freedom in such a way as to make its effect on the satisfaction with freedom more positive the more social trust there is.^11^

Why so? We propose at least three mechanisms: behavioral changes in the market and civil society; better governance in the public sector; and higher economic growth. The first mechanism, inspired by Rothstein (2000), starts from the realization that mere legal freedom may not suffice for a rich, vibrant, satisfying culture of choice. In addition, people might have to trust others in order for them to initiate interaction and exchange that result in their goals being met more successfully. The more they trust others, they expect them not to take advantage of them, not to exploit them, not to behave opportunistically towards them – and they will therefore engage with others in a cooperative, responsible and respectful fashion, generating more satisfaction with the legal freedom that underlies a system of social interaction.^12^

The second mechanism, inspired by Bjørnskov (2010), notes that the way the public sector functions is not only a result of the legal rules in place but also dependent on the culture. In a country in which people, in addition to having a high-quality legal system, trust others, the quality of governance is higher, implying, e.g., less corruption, more efficient handling of various errands and non-discrimination, and more careful protection of citizens’ rights. This will in turn facilitate any type of cooperative venture that involves the public sector, and it will therefore make it easier for many to fulfill their ambitions, resulting in satisfaction with freedom of choice.

The third mechanism, inspired by Knack and Keefer (1997), starts from the documented finding that social trust leads to higher economic growth. In the presence of social trust, the effect on growth of the legal system can be expected to be even higher. For freedom of choice to result in satisfaction, it is important that the games being played between people are not of a zero-sum nature—and as argued by Friedman (2005), avoiding such a situation is indeed an important “moral” consequence of growth. An increasing pie makes conflict less probable and productive cooperation, resulting in satisfaction with freedom of choice, more likely.

We consider two further potential moderators: democracy and national income. As for democracy, it may be conducive to satisfaction with freedom that individuals can rationally expect that their rights are continually protected and cannot be removed, ignored or derogated on a whim. In other words, as argued by, e.g., Keefer and Stasavage (2003) in the context of monetary institutions and Justesen and Kurrild-Klitgaard (2013) in the context of property rights institutions, sufficiently strong democratic veto institutions may be necessary for people to make beneficial long-term choices, suggesting a positive interaction effect from democracy. As for average income, for legal freedom to affect individuals’ life choices, it may also be necessary that they have the material resources to achieve their goals. This aspect is for example central to Sen’s (1993) capabilities approach, which argues for complementarity between the negative freedoms inherent in legal freedom and the positive freedom associated with access to resources.

In summary, the association between legal freedom and satisfaction with freedom is theoretically ambiguous but is likely influenced by social trust, democracy and income.

## Data and empirical approach

### Data

Our dependent variable is from Gallup (2020) and is the share of people in a country who reply “satisfied” to the question “Are you satisfied or dissatisfied with your freedom to choose what you do with your life?”. Gallup uses probability-based sampling to obtain nationally representative samples of residents aged 15 and older. It uses telephone interviewing, except in countries with relatively poor telephone coverage, where face-to-face interviews are held. In most countries, the sample consists of 1000 people, except in very large countries such as Russia, where the sample size is doubled. In a few very small countries, such as Iceland, sample sizes can be slightly smaller than 1000.

We understand this question as being a cognitive evaluation of the level of freedom of choice faced by the respondent, where satisfaction indicates that the individual is (more or less) satisfied with the level of freedom of choice they have got and where dissatisfaction indicates that the individual is (more or less) dissatisfied with it, either wanting more or less (for reasons discussed in the theory section). On the country level, a high share of satisfied respondents thus indicates that most people more or less find the present level of freedom of choice to be in line with their preferences.^13^ This measure has not been used in the subjective well-being literature very much, and, to our knowledge, not in relation to legal freedom.^14^

Our main explanatory variables are seven indicators of legal freedom from the Human Freedom Index (Vásquez & McMahon, 2020): the rule of law; security and safety; freedom of movement; freedom of religion; freedom of association, assembly and civil society; freedom of expression and information; and freedom of identity and relationships. These indicators capture different aspects of freedom of choice, and Vásquez and McMahon (2020, pp. 10–11) offer this overall interpretation: “This index is thus an attempt to measure the extent to which the negative rights of individuals are respected in the countries observed. By negative rights, we mean freedom from interference—predominantly by government—in people’s right to choose to do, say, or think anything they want, provided that it does not infringe on the rights of others to do likewise.” Each indicator is reported on a scale from 0 to 10, where 10 is the maximum freedom, and each indicator is based on a number of further variables, specified in Table 3 in the Appendix. These are, in turn, collected from external data sources and are, as a rule, based on assessments by national experts.^15^

We use the following control variables, based on established practice in the cross-country life-satisfaction literature: confidence in government, social trust, democracy (Dorn et al., 2007, find a positive effect of democracy on happiness), log GDP per capita, trade and government spending. Confidence in government is also drawn from Gallup (2020), which asks respondents whether or not they have confidence in their national government. Social trust is measured, as is standard, as the share of respondents stating that most people can be trusted, which we derive from the World Values Survey and the regional barometer surveys (cf. Bjørnskov, 2011). The log to real purchasing-power adjusted GDP per capita, trade volumes and government final consumption spending (both in percent of GDP) are from the Penn World Tables, mark 10 (Feenstra et al., 2015). We measure democracy by the minimalist dichotomous indicator in Bjørnskov and Rode (2020) such that democracy does not conceptually overlap with any of our measures of human freedom. Finally, we add a set of fixed effects for years and eight broad world regions.

The sample with full data consists of 133 countries across the world, and the analysis covers the period for which key data are available, 2008–2018. Descriptive statistics are presented in Table 4 in the Appendix. The range of the dependent variable is between a low of 0.26 (Bosnia and Herzegovina in 2009) and a high of 0.98 (Uzbekistan in 2017). Among democracies, for which we are certain that the surveys were not doctored, the range is between 0.26 (Burundi in 2008) and 0.96 (Denmark in 2019).

### Empirical approach

Our empirical approach is dictated by the data that form a highly unbalanced panel. As is the case for data on life satisfaction, the data on satisfaction with the freedom to make life choices are strongly persistent over the 11-year time period for which we have data. This persistence prevents us from using a fixed effects estimator, as country fixed effects would capture the time-invariant part of our main variables and thus most of the relevant variation. Instead, we employ a random effects estimator with fixed effects for years and eight broad world regions: the Caucasus and Central Asia, East Asia, Eastern Europe, Latin American and the Caribbean, the Middle East and North Africa, South Asia, Sub-Saharan Africa, and Western Europe and the European offsprings in North America and Oceania.

In subsequent tests, we introduce a set of interactions between our legal freedom factors and social trust, democracy and the log to GDP per capita. For all of these interactions, we interpret the results with the proper conditional standard errors clustered at the country level and provide interaction plots for those that are robustly significant (Brambor et al., 2006).

For identification, the regular approach in the absence of quasi-natural experiments and differences is to apply instrumental variables. However, after a thorough search for viable instruments, we have found no candidates that were obviously valid and provided identification for our legal freedom variables. A major challenge is that the instruments must not only be valid and sufficiently strong—they must also be specific to each of the seven measures of legal freedom. Our best bet was a spatial lag (the average value of neighboring countries), which nevertheless proved to be weak and very noisy. In the process, we have also noted that certain variables used in previous research, such as genetic diversity and legal origins, provide very little identification in the present sample.

While we therefore acknowledge that we cannot with any certainty establish causality—it remains possible that respondents’ subjective freedom of choice reflects some factor that affects some or all of our measures of legal freedom—the structure of the potential heterogeneity of effects, as revealed in the interaction analysis, may provide some information about the degree to which the overall associations are endogenous (Dreher et al., 2018). The potential endogeneity bias inherent in our approach—which would occur if the satisfaction with freedom or some highly correlated other aspect of individual beliefs causally affects legal freedom—is not clearly signed. On the one hand, it is possible that individuals who are more satisfied with freedom are more likely to push politically for more legal freedom. On the other hand, it is possible that people that are less satisfied with freedom are more likely to demand more legal freedom in an attempt to deal with a cause of their dissatisfaction. Practically, we therefore cannot do anything about the problem but merely note that any bias will cause our estimates to be less precise.

## Results

### Baseline regression results

We present the results of our baseline specifications in Table 1. Each column contains one indicator of legal freedom.

**Table 1:** Legal freedom and satisfaction with freedom of choice

	1	2	3	4	5	6	7
Confidence in government	0.276*** (0.029)	0.274*** (0.029)	0.274*** (0.029)	0.279*** (0.029)	0.288*** (0.029)	0.276*** (0.029)	0.273*** (0.028)
Social trust	− 0.026 (0.077)	− 0.009 (0.077)	− 0.006 (0.078)	− 0.009 (0.078)	0.011 (0.075)	− 0.005 (0.077)	− 0.015 (0.077)
Democracy	0.004 (0.012)	0.006 (0.012)	0.005 (0.012)	0.011 (0.012)	− 0.009 (0.012)	0.004 (0.011)	0.004 (0.011)
Log GDP per capita	0.051*** (0.013)	0.057*** (0.011)	0.057*** (0.011)	0.060*** (0.011)	0.060*** (0.011)	0.057*** (0.011)	0.057*** (0.013)
Trade	− 0.001 (0.015)	0.001 (0.015)	0.001 (0.015)	− 0.005 (0.014)	− 0.007 (0.014)	0.001 (0.015)	0.001 (0.015)
Government spending	− 0.041 (0.100)	− 0.046 (0.103)	− 0.049 (0.102)	− 0.069 (0.097)	− 0.077 (0.101)	− 0.047 (0.099)	− 0.025 (0.104)
Rule of law	0.009 (0.008)						
Safety and security		0.002 (0.005)					
Freedom of movement			0.002 (0.002)				
Religious freedom				− 0.001 (0.005)			
Freedom of assembly					0.008** (0.003)		
Freedom of expression						0.005 (0.005)	
Freedom of identity							0.006* (0.003)
Region FE	Yes	Yes	Yes	Yes	Yes	Yes	Yes
Year FE	Yes	Yes	Yes	Yes	Yes	Yes	Yes
Observations	1204	1204	1204	1229	1228	1228	1204
Countries	132	132	132	133	133	132	132
R^2^	0.564	0.556	0.554	0.552	0.559	0.559	0.559
Wald Chi sq	615.46	610.78	622.69	619.00	614.33	636.48	590.91

*** (**) [*] indicate significance at p < 0.01 (p < 0.05) [p < 0.10]. Numbers in parentheses are standard errors clustered at the country level. All estimates are obtained with a random effects estimator including a constant term

First, when looking at the control variables, we find that two of them matter for satisfaction with freedom of choice: confidence in government and log GDP per capita. People in countries where the government is considered trustworthy, and people in richer countries, are more satisfied with freedom of choice. This is not surprising—confidence in government implies fair and effective governance, which facilitates the fulfilment of one’s ambitions in life, and higher national income implies more resources to realize one’s goals (Helliwell & Huang, 2008; Stevenson & Wolfers, 2008). Excluding the region fixed effects reduces the R^2^ by about 0.1; the year fixed effects provide slightly more identification. As such, the fairly similar precision of the specification across Tables 1–4 is not caused by the fixed effects swamping any other factors. It also bears noting that when adding the control variables gradually, this has a minimal effect on the point estimates.

When looking at our seven indicators of legal freedom, we see that two are related to satisfaction with freedom of choice in a statistically significant and positive way: freedom of association, assembly and civil society; and freedom of identity and relationships. The more legal freedom in these areas, which we interpret as more freedom of choice being introduced, the more likely it is that people are satisfied with the freedom of choice they face. This suggests non-saturation, that the freedom of choice actually faced by people before an increase did not exhaust satisfaction with freedom of choice. Thus, we can interpret the positive sign such that people have a preference for more freedom of choice and that it can be (at least partly) satisfied by more legal freedom expanding freedom of choice. Still, for freedom of identity and relationships, statistical significance is rather weak and not robust to removing potential outliers; and neither freedom of association, assembly and civil society or freedom of identity and relationships are robust to adding three cultural indicators from the Hofstede et al. (2010) dataset (power distance, individualism and uncertainty avoidance), or to removing all control variables.^16^ This altogether indicates that legal freedom per se does not seem able to generate general and strong satisfaction with freedom of choice. This conclusion is further reinforced by considering the size effects, which are small. For example, increasing freedom of assembly by 5 units (half of the entire index scale) implies an increase in the probability of being satisfied with freedom of 4 percentage points.^17^

### Interaction with social trust

As suggested by existing studies and hypothesized in Sect. 2, it could nevertheless be the case that the formal institutions captured by legal freedom need something else in order to generate satisfaction with freedom of choice, viz., a certain cultural context characterized by social trust. We investigate this in Table 5 in the Appendix, where social trust and the indicators of legal freedom are interacted with each other.

There are indications of interaction effects for two indicators of legal freedom: the rule of law; and freedom of identity and relationships. However, the interpretation of interaction terms per se is complicated by two factors: first, that the significance of the interaction terms indicates whether a one-point change in the interacting variable significantly changes the relation, and second, that some interactions may be driven by obvious outliers. The first complication means that we can still obtain significant results even if the interaction term does not appear significant when the confidence interval is too wide for a relatively small change in social trust to yield a significantly different estimate of legal freedom. The second complication derives from the fact that the indicators of legal freedom are censored (by the ten-point scale), and some distributions are heavily skewed. This is a major concern in a number of cases where a substantial part of the observations has a perfect rating of 10 in, e.g., the assessment of freedom of identity.

Since the table for these reasons does not reveal the full conditional effects, for a more granular analysis, we turn to a marginal plot. The particular relationship illustrated in the figure was chosen because it is the only one, out of the seven, that displays a statistically significant interaction term for a segment of social trust and that is not sensitive to the removal of outliers at the top or bottom.^18^

**Fig. 1:**
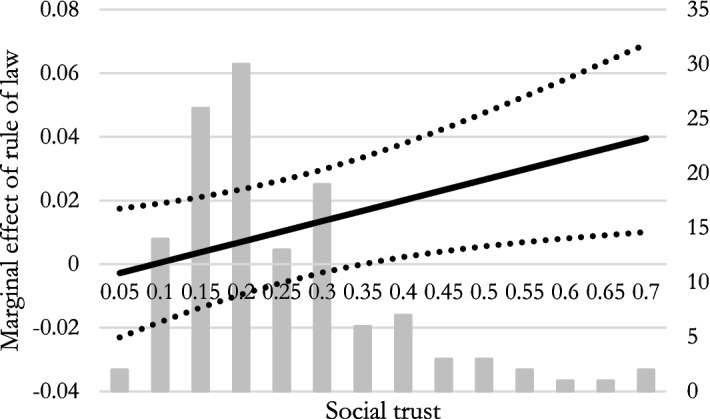
Conditional effects when interacting the rule of law and social trust. The dotted lines show the 95% confidence interval. Grey columns illustrate how many countries within the sample are in each “bin” of social trust

Figure 1 shows how the marginal effect of the rule of law varies with social trust. Interestingly, we find a significant interaction effect (at the 5% level) above trust levels of about 0.33; as indicated by the grey columns in the figure, this condition holds for about 30 of the 133 countries. When a larger share than that are trusting others in a country, the effect of the rule of law on satisfaction with freedom of choice receives a boost, and increases with social trust. Going from a trust level of 0.33 to one of 0.67, i.e., doubling the amount of trust and moving from a Spanish to a Nordic level of trust, also doubles the marginal effect of the rule of law.

For a better understanding of the size effect, consider the following. At a level of social trust around 40%—approximately that of Western Germany and the United Kingdom—a one deviation shock to the rule of law yields an increase in satisfaction with freedom of 22% of a standard deviation. Illustrated in another way, the substantially better rule of law in Estonia, compared to its two Baltic neighbors Latvia and Lithuania, can explain three fourths of the difference (a 26% difference in freedom) in terms of satisfaction with freedom. As such, although the absolute numbers may appear small, changes in rule of law in societies with average to high levels of social trust are meaningful and politically significant.

We thereby see that social trust performs as hypothesized: it complements and boosts the positive effect of legal freedom on satisfaction with the freedom of choice people perceive they have.

### Interactions with democracy and GDP per capita

To further investigate the potential heterogeneity of effects of legal freedom, we conduct interaction tests with democracy and average national income. Democracy is relevant since it is perceivable that legal freedom makes people satisfied with freedom of choice differently depending on what the basic system of government is. It is also conceivable that the survey data, which we rely on here, are substantially less subject to respondent bias or government interference in democracies, and thus more precise. Finally, as hypothesized above, the existence of democratic veto institutions may be a necessary condition for legal freedom to clearly affect citizens’ long-term life choices and, thereby, their satisfaction with freedom of choice.

As Table 2 indicates, the only area where an effect can be detected is for freedom of association, assembly and civil society. Since democracy is a dummy variable, we cannot produce a meaningful marginal plot. However, following Brambor et al. (2006) and calculating marginal effects, the point estimates suggest that democracy substantially increases the effect of freedom of association, assembly and civil society, approximately tripling its size and making it statistically significant. While the estimate in autocracies is 0.005, the corresponding estimate in democracies is 0.005 + 0.011 = 0.016, which is strongly significant (p < 0.01). In other words, democracy appears a necessary condition for the right to associate and assemble freely to positively affect freedom of choice.^19^ We also note that when employing the measure of the strength of veto institutions developed by Henisz (2002), instead of democracy, we find the same result: the association with the freedom of association, assembly and civil society only becomes statistically significant above a level of about 0.3, or about the minimum level of veto institutions observed in stable democracies.^20^

**Table 2:** Interaction between legal freedom and democracy

	1	2	3	4	5	6	7
Democracy	− 0.036 (0.056)	0.032 (0.057)	0.015 (0.027)	0.016 (0.054)	− 0.075* (0.044)	− 0.029 (0.065)	0.018 (0.028)
Rule of law	0.002 (0.013)						
Safety and security		0.004 (0.007)					
Freedom of movement			0.003 (0.003)				
Religious freedom				− 0.000 (0.006)			
Freedom of assembly					0.005 (0.004)		
Freedom of expression						0.003 (0.008)	
Freedom of identity							0.007** (0.003)
Freedom* democracy	0.009 (0.012)	− 0.004 (0.007)	− 0.001 (0.004)	− 0.001 (0.008)	0.011* (0.006)	0.004 (0.009)	− 0.002 (0.004)
Region FE	Yes	Yes	Yes	Yes	Yes	Yes	Yes
Year FE	Yes	Yes	Yes	Yes	Yes	Yes	Yes
Observations	1204	1204	1204	1229	1228	1228	1204
Countries	132	132	132	133	133	132	132
R^2^	0.569	0.555	0.554	0.553	0.570	0.561	0.556
Wald Chi sq	660.42	616.38	627.82	629.66	631.69	652.44	593.46

*** (**) [*] indicate significance at p < 0.01 (p < 0.05) [p < 0.10]. Numbers in parentheses are standard errors clustered at the country level. All estimates are obtained with a random effects estimator including a constant term. For reasons of space, all control variables are not reported

**Fig. 2:**
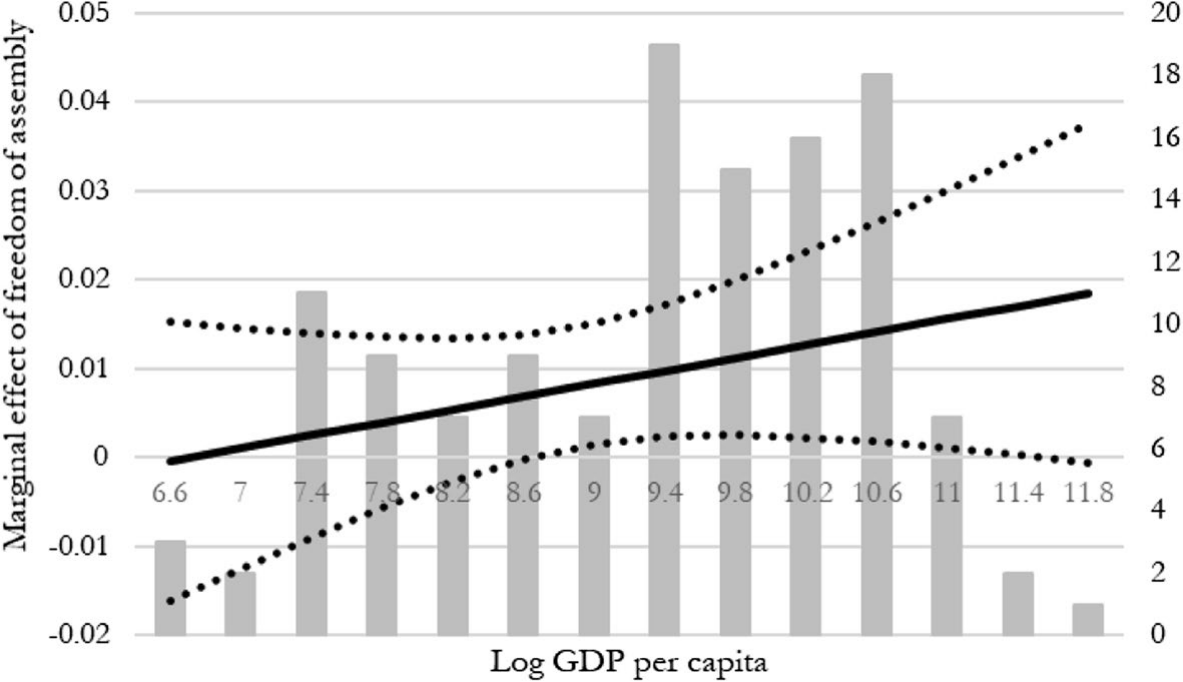
Conditional effects when interacting freedom of assembly and log GDP per capita. The dotted lines show the 95% confidence interval. Grey columns illustrate how many countries within the sample are in each “bin” of GDP per capita

We next turn to an interaction between GDP per capita and legal freedom, since it is conceivable that satisfaction with freedom of choice is larger if more legal freedom is accompanied by more material resources enabling more actually preferred choices. In other words, the results test whether there is complementarity between negative and positive freedom in the sense of Berlin (1969).^21^ The results, reported in Table A4 in the Appendix, indicate that there are significant interaction effects for two indicators of legal freedom. However, as the result pertaining to safety and security turns out to be driven entirely by outlier observations, we proceed to further analyze the marginal plot for the single relationship that displays statistical significance and that is robust to outliers, in Fig. 2.^22^

Figure 2 shows that above a log GDP per capita value of about 8.6, which corresponds to 5400 USD in 2011 US prices, higher national income increases the effect of freedom of association, assembly and civil society on satisfaction with freedom of choice, in line with our expectation.^23^ Yet, the conditional relationship is borderline significant and should be interpreted with care for that reason. One should also be careful, as the majority of rich countries are also stably democratic, and we therefore cannot clearly distinguish between living in a wealthy and living in a stably democratic nation.

These further interaction exercises show that the effect of legal freedom on satisfaction with freedom of choice is not generally or strongly influenced by the form of government or GDP per capita; but for freedom of association, assembly and civil society, more resources and democratic institutions do imply that this form of satisfaction increases.^24^

Overall, we thus find evidence that the effects of legal freedom on the satisfaction with freedom of choice—when we observe any effects—tend to be mediated by either informal institutions, democracy or income. The effects of rule of law are moderated by social trust, while the particular effects of freedom of assembly appear to be moderated by democracy and income.^25^

### Alternative measures of the rule of law

Lastly, we have tested how robust the rule of law results are to using two alternative measures. The first, judicial accountability, is from the Varieties of Democracy or V-Dem project (Coppedge et al., 2020) and captures the de facto degree to which there are specific and effective procedures for disciplining and removing misbehaving (often corrupt or politically motivated) judges in order to keep the judicial system effective and fair. The second, the WGI rule of law index is from the Worldwide Governance Indicators (Kaufmann et al., 2010). It captures the de facto integrity and fairness of the institutions protecting contract enforcement and property rights, which ensures the quality of the police and courts. We note that while the former captures rather different features, not least reflecting that existing legislation is effectively implemented whatever its content, the findings when applying the latter are qualitatively similar to our other findings. See Table A5 in the Appendix for details.

## Concluding remarks

Freedom of choice is a highly valued feature of life for many, and it is guaranteed through legal freedom, i.e., the rule of law and civil rights. However, we know little about whether people find satisfaction in the freedom of choice they have. This study sheds light on that issue.

Theoretically, it is not clear what the relationship looks like. What speaks in favor of a positive effect is that legal freedom allows people to make the choices they want in life (so long as they do not violate a similar right by others). This prevents obstacles when people realize their ambitions and makes it more likely that these ambitions will be met, resulting in satisfaction with freedom of choice. Still, the relationship can be weak or negative. This can happen if people do not reach the goals they set out to reach—after all, legal freedom only removes obstacles and does not provide resources or capabilities. It can also happen if people observe others behaving opportunistically or exploitatively, using their freedom of choice to treat others badly in pursuit of narrow personal gain, or if people feel overburdened by the freedom of choice accorded them by legal freedom. It also remains possible that the value of specific legal freedoms is substantially larger for minorities than the broad population (cf. Berggren et al., 2017), which can explain why they do not turn out to be important for satisfaction with freedom for the larger population.

We suggest that social trust is a potentially important factor that, when interacted with legal freedom, can turn the effect of legal freedom on satisfaction with freedom of choice more positive. The idea is that choice is not executed in a cultural vacuum but is the result of both formal institutions and culture, in this case the degree to which people trust each other. When they do, they expect others to behave more cooperatively, which stimulates more interaction and “gains from trade”, resulting in higher satisfaction. Likewise, the material resources at hand and the political system may influence how legal freedom affects satisfaction with freedom.

In our empirical study, using panel data for up to 133 countries during the period 2008–2018, we identify only two out of seven indicators of legal freedom as positively related to satisfaction with freedom of choice in our baseline analysis: freedom of association, assembly and civil society, and freedom of identity and relationships. However, these findings are not robust to outliers and changes in the model specification. In contrast, we do find certain robust results in our interaction analysis—for specific indicators and over and above certain threshold values of the interaction variables. When interacting the seven indicators with social trust, we find that the importance of the rule of law is increasing in social trust. This indicates that certain formal institutions, in our case the rule of law, and culture, in the form of social trust, are complementary in the production of satisfaction with freedom of choice. Interacting our indicators of legal freedom with democracy show that democracy substantially increases the influence of freedom of association, assembly and civil society. Interactions with GDP per capita indicate that freedom of association, assembly and civil society generates more satisfaction with freedom, and the more so the more resources they have.^26^ Even though findings for freedom of identity and relationships are sensitive to potential outliers in the linear specifications, it bears mentioning that they are robust when using logarithmic transformations, suggesting that this factor may be taken to be positively related to satisfaction with freedom as well.

However, we do not want to overemphasize the separability of these interaction results, since the moderators tend to be correlated: it is often the same group of countries that are characterized by high incomes, stable democracy and relatively trusting populations. This may be taken to indicate that some broadly defined aspect of modernity moderates the effects.

It is in any case noteworthy that few indicators of legal freedom can be shown to relate to satisfaction with freedom of choice. Yet, our findings suggest that those who do wish to increase satisfaction with freedom of choice through more legal freedom would do well to consider the material circumstances in society, the political system as well as the cultural embeddedness of legal freedom. For example, it may not suffice to strengthen and reform the formal institutions producing the rule of law to make freedom of choice a truly satisfactory experience for citizens—it may take social trust to make this outcome likely. Of course, changing the culture of a society is easier said than done, but previous research suggests that reduced inequality (Jordahl, 2009), less corruption (You, 2018) and a stronger rule of law (Berggren & Jordahl, 2006; Cassar et al., 2014) might be avenues worth trying, for this reason and others.

## Data Availability

Will be made available upon the request of any researcher.

## Appendix

See Tables 3﻿, 4﻿, 5﻿, 6 and 7﻿.

**Table 3:** Indicators of legal freedom

1. Rule of law
1.1. Procedural justice
1.2. Civil justice
1.3. Criminal justice
2. Security and safety
2.1. Homicide
2.2. Disappearances, conflict, and terrorism
2.2.1. Disappearances
2.2.2. Violent conflicts
2.2.3. Organized conflicts
2.2.4. Terrorism fatalities
2.2.5. Terrorism injuries
2.3. Women’s security and safety
2.3.1. Female genital mutilation
2.3.2. Missing women
2.3.3. Inheritance rights
2.3.3.1. Widows
2.3.3.2. Daughters
3. Movement
3.1. Domestic movement
3.2. Foreign movement
3.3. Women’s movement
4. Religion
4.1. Establishing and operating religious organizations
4.2. Harassment and physical hostilities
4.3. Legal and regulatory restrictions
5. Association, assembly and civil society
5.1. Association
5.2. Assembly
5.3. Establishing and operating political parties
5.4. Establishing and operating professional organizations
5.5. Establishing and operating educational, sporting, and cultural organizations
6. Expression and information
6.1. Press killed
6.2. Press jailed
6.3. Laws and regulations that influence media content
6.4. Political pressures and controls on media content
6.5. Access to cable/satellite
6.6. Access to foreign newspapers
6.7. State control over internet access
7. Identity and relationships
7.1. Legal gender
7.2. Parental rights
7.2.1. In marriage
7.2.2. After divorce
7.3. Same-sex relationships
7.3.1. Male-to-male relationships
7.3.2. Female-to-female relationships
7.4. Divorce

Source: Vásquez and McMahon (2020, p. 15)

**Table 4:** Descriptive statistics

	Mean	Standard deviation	Observations
Satisfaction with freedom to make life choices	0.738	0.143	1706
Social trust	0.245	0.125	1858
Rule of law	5.271	1.563	1614
Safety and security	8.109	1.483	1614
Freedom of movement	7.778	2.632	1614
Religious freedom	7.382	1.669	1683
Freedom of assembly	7.181	2.323	1672
Freedom of expression	8.379	1.389	1614
Freedom of identity	7.189	3.212	1614
Confidence in government	0.483	0.193	1522
Democracy	0.591	0.492	2076
Log GDP per capita	9.188	1.202	1872
Trade	0.637	0.546	1872
Government spending	0.180	0.074	1872

**Table 5:** Interaction between legal freedom and social trust

	1	2	3	4	5	6	7
Social trust	− 0.004** (0.002)	− 0.004 (0.003)	0.001 (0.001)	− 0.003 (0.002)	0.000 (0.002)	0.001 (0.004)	− 0.006*** (0.002)
Rule of law	− 0.006 (0.012)						
Safety and security		− 0.009 (0.009)					
Freedom of movement			0.005 (0.004)				
Religious freedom				− 0.012 (0.009)			
Freedom of assembly					0.008 (0.007)		
Freedom of expression						0.009 (0.012)	
Freedom of identity							− 0.008* (0.005)
Freedom* trust	0.001** (0.000)	0.000 (0.000)	− 0.000 (0.000)	0.000 (0.000)	− 0.000 (0.000)	− 0.000 (0.000)	0.001*** (0.000)
Region FE	Yes	Yes	Yes	Yes	Yes	Yes	Yes
Year FE	Yes	Yes	Yes	Yes	Yes	Yes	Yes
Observations	1204	1204	1204	1229	1228	1204	1204
Countries	132	132	132	133	133	132	132
R^2^	0.574	0.561	0.553	0.549	0.559	0.559	0.567
Wald Chi sq	658.04	649.47	649.03	605.86	628.85	652.67	618.12

*** (**) [*] indicate significance at p < 0.01 (p < 0.05) [p < 0.10]. Numbers in parentheses are standard errors clustered at the country level. All estimates are obtained with a random effects estimator including a constant term. For reasons of space, all control variables are not reported

**Table 6:** Interaction between legal freedom and log GDP per capita

	1	2	3	4	5	6	7	8
Log GDP per capita	0.062** (0.029)	0.131*** (0.033)	0.063*** (0.014)	0.055** (0.027)	0.036 (0.023)	0.079** (0.037)	0.068*** (0.016)	0.125*** (0.031)
Rule of law	0.029 (0.058)							
Safety and security		0.086** (0.039)						
Freedom of movement			0.009 (0.015)					
Religious freedom				− 0.006 (0.033)				
Freedom of assembly					− 0.024 (0.028)			
Freedom of expression						0.030 (0.038)		
Freedom of identity							0.019 (0.019)	
Freedom from regulation								0.109*** (0.042)
Freedom* log GDP	− 0.002 (0.006)	− 0.009** (0.004)	− 0.001 (0.002)	0.001 (0.004)	0.004 (0.003)	− 0.003 (0.004)	− 0.002 (0.002)	− 0.011** (0.005)
Region FE	Yes	Yes	Yes	Yes	Yes	Yes	Yes	Yes
Year FE	Yes	Yes	Yes	Yes	Yes	Yes	Yes	Yes
Observations	1204	1204	1204	1229	1228	1204	1204	1288
Countries	132	132	132	133	133	132	132	133
R^2^	0.563	0.559	0.554	0.553	0.562	0.554	0.559	0.563
Wald Chi sq	628.90	673.25	643.52	620.43	599.83	643.86	585.80	833.57

*** (**) [*] indicate significance at p < 0.01 (p < 0.05) [p < 0.10]. Numbers in parentheses are standard errors clustered at the country level. All estimates are obtained with a random effects estimator including a constant term. For reasons of space, all control variables are not reported

**Table 7:** Interaction between legal freedom and democracy—using the V-Dem index of judicial accountability and the WGI Rule of Law index

	1	2	3	4	5	6	7	8
*Compare to*	Table 1, col. 1	Table 5, col. 1	Table 2, col. 1	Table 6, col. 1	Table 1, col. 1	Table 5, col. 1	Table 2, col. 1	Table 6, col. 1
Confidence in government	0.288*** (0.028)	0.288*** (0.028)	0.289*** (0.028)	0.288*** (0.028)	0.283*** (0.027)	0.284*** (0.028)	0.284*** (0.027)	0.281*** (0.027)
Social trust	− 0.000 (0.001)	− 0.000 (0.001)	− 0.000 (0.001)	0.000 (0.001)	− 0.000 (0.001)	− 0.001 (0.001)	− 0.000 (0.001)	− 0.000 (0.001)
Democracy	0.014 (0.011)	0.014 (0.011)	− 0.013 (0.029)	0.014 (0.011)	0.008 (0.011)	0.008 (0.011)	0.012 (0.014)	0.007 (0.011)
Log GDP per capita	0.061*** (0.012)	0.059*** (0.012)	0.059*** (012)	0.084*** (0.020)	0.037** (0.015)	0.035** (0.014)	0.038** (0.015)	0.034** (0.015)
Trade	− 0.008 (0.014)	− 0.007 (0.013)	− 0.005 (0.013)	− 0.006 (0.013)	− 0.014 (0.013)	− 0.014 (0.013)	− 0.013 (0.014)	− 0.012 (0.013)
Government size	− 0.071 (0.096)	− 0.072 (0.095)	− 0.077 (0.092)	− 0.080 (0.096)	− 0.079 (0.091)	− 0.076 (0.091)	− 0.082 (0.091)	− 0.092 (0.089)
Judicial accountability	0.007 (0.009)	0.004 (0.017)	− 0.004 (0.016)	0.109 (0.075)				
WGI Rule of Law					0.047*** (0.015)	0.033 (0.022)	0.042** (0.020)	0.109 (0.072)
Accountability* trust		0.000 (0.001)						
Rule of law* trust						0.001 (0.001)		
Accountability* democracy			0.015 (0.015)					
Rule of law* democracy							0.007 (0.016)	
Accountability* GDP				− 0.011 (0.008)				
Rule of law* GDP								− 0.007 (0.008)
Region FE	Yes	Yes	Yes	Yes	Yes	Yes	Yes	Yes
Year FE	Yes	Yes	Yes	Yes	Yes	Yes	Yes	Yes
Observations	1320	1320	1320	1320	1322	1322	1322	1322
Countries	133	133	133	133	134	134	134	134
R squared	0.556	0.559	0.566	0.549	0.578	0.584	0.578	0.577
Wald Chi sq	664.32	673.64	696.62	719.63	775.71	816.21	810.33	824.38

*** (**) [*] indicate significance at p < 0.01 (p < 0.05) [p < 0.10]. Numbers in parentheses are standard errors clustered at the country level. All estimates are obtained with a random effects estimator including a constant term. For reasons of space, all control variables are not reported

## Footnotes

1 Zakaria (1997, p. 22) defines liberal democracy as “a political system marked not only by free and fair elections, but also by the rule of law, a separation of powers and the protection of basic liberties of speech, assembly, religion and property.” Cf. Mukand and Rodrik (2020).

2 We henceforth refer to this kind of freedom as “legal freedom”, indicating that it is provided through legal rules (i.e., formal institutions in the sense of North, 1990). It is thus a type of negative freedom (Berlin, 1969).

3 To situate this study, it may be useful to clarify what we mean by certain key terms. We regard *subjective well-being* as the most comprehensive term for denoting how people subjectively experience their lives. Following Diener et al. (2009), a person’s subjective well-being consists of two elements: their cognitive and affective evaluations of their life. We refer to the first part, cognitive evaluations, as *life satisfaction*, and it concerns a person’s considered view of life as a whole, distinct from temporary emotional states. The second part, affective evaluations, are referred to as *happiness* (if positive; if negative: unhappiness). Happiness and unhappiness are distinct from each other and are emotion-based assessments of how one experiences life at a particular point in time. While our dependent variable of interest, satisfaction with freedom, is conceptually distinct from life satisfaction, we interpret the underlying reason for being satisfied with freedom of choice that it allows people to make choices that contribute to more life satisfaction (even though there are other possible bases for valuing freedom of choice).

4 For a study of the institutional determinants of these indicators of legal freedom, see Berggren and Gutmann (2020).

5 We do not, however, explore the importance of broader governance indicators or economic freedom. Helliwell and Huang (2008), Ott (2011), Knoll et al. (2013), Rode (2013) and Graafland and Compen (2015) are among the many studies exploring the consequences on subjective well-being of such factors.

6 Social trust has been found to be positive for life satisfaction – see, e.g., Diener and Suh (1999), Helliwell (2003, 2006), Bjørnskov et al., (2007, 2010), Bjørnskov (2008), Kroll (2008), Ram (2010), Growiec and Growiec (2014), Rodríguez-Pose and von Berlepsch (2014), Puntscher et al. (2015), Hommerich and Tiefenbach (2018) and Majeed and Samreen (2020).

7 Some studies have life satisfaction as the dependent variable, others happiness, but none seems to have ours (satisfaction with freedom). The terminology used for the explanatory variable of main interest varies, the most common ones being civil rights, civil liberties, human rights and freedom, but they are all conceptually close.

8 While the concept of satisfaction with freedom has not been used in much empirical research, it is related to psychological concepts such as individuals’ locus of control and psychological autonomy (see Sect. 2.1 for related literature).

9 We recognize that there are other possible interpretations of what underlies a reply to the Gallup question. For example, a deontologist would hold that a person being dissatisfied with the freedom of choice he or she actually has is dissatisfied because that person values (more) freedom of choice per se, for intrinsic reasons. However, it does not matter for our results, their interpretation or importance what the underlying reason for valuing freedom of choice is.

10 See Schwarz et al. (2002) for a study indicating that choice can feel worse off with a large number of choice options. Yet, Scheibehenne et al. (2010) do not find robust evidence of such an effect of “choice overload”.

11 This finding is in line with the recent research of McCannon et al. (2018) and Bartling et al. (2021), who show that trust and contract enforcement are complements. However, neither study includes satisfaction as an outcome variable, as we do. Our study can also be compared to Williamson and Mathers (2011), who find that both culture (in the form of individualism vs. collectivism) and economic freedom influence economic growth, but as substitutes; to Bjørnskov (2011), who reports that institutional quality is more effective against corruption if there is high social trust; to Graafland and Compen (2015), who demonstrate an interaction effect between social trust and economic freedom on life satisfaction; and to Graafland (2020), who shows the same with the Human Development Index as the outcome variable. More generally, Alesina and Giuliano (2015) present an overview of the emerging research field linking culture and institutions to each other and to various outcomes, to which this study is a contribution.

12 On how social trust stimulates cooperative behavior, see Gächter et al. (2004) and Balliet and van Lange (2013).

13 It does not matter for our empirical analysis as such what the underlying reason for why respondents to the Gallup question reply one way or the other. While we take it to be a desire for life satisfaction – wanting a level of freedom that allows one to make choices to achieve such satisfaction – it is possible to interpret the underlying reason in other ways. For example, a deontologist could value freedom of choice intrinsically, disregarding any consequences, e.g., in terms of subjective well-being, of such freedom.

14 While we cannot dismiss the possibility that the measure captures factors related to freedom from social pressure rather than legal freedom, it appears to be a minor problem. For example, our measure is not related to Gutmann and Voigt’s (2022) data on Todd’s family types.

15 As pointed out by Chilton and Versteeg (2020), enshrining rights in constitutions does not automatically ensure that those rights will be respected in practice, which speaks in favor of using this kind of de facto rather than *de jure* indicators of legal freedom.

16 However, these two exercises do not induce non-robustness in the ensuing interaction analysis. We have also performed a robustness test regarding functional form, in three ways: adding quadratic terms to the indicators of legal freedom, taking the logarithm of these indicators and taking the logarithm of these indicators and the dependent variable. The quadratic terms turn out to add noise – they do not provide a better fit than the linear baseline specification of Table 1. However, the results of the two logarithmic exercises are comparable to the linear results, the main difference being that the point estimate for freedom of identity and relationships is robust to the removal of potential outliers. Results are available on request.

17 In addition, we have performed a set of Tobit estimates to handle the censoring problem in our data. We do not present these results here, as they are almost identical to the results in Table 1. However, these estimates are available on request.

18 More specifically, our outlier test has been applied to all the significant results in the interaction analysis by removing observations with the five percent largest and smallest scores for the variables of interest, as well as the dependent variable.

19 In addition, the small and generally insignificant interactions and the varying sign of the interactions indicate that any causality problem is likely to be relatively small. Had causality been a major issue, such that freedom of choice affected legal freedom through the political process, we would have expected to observe substantial, significant and positive interactions throughout.

20 These results are not shown here but are available on request.

21 Cf. Sen’s (1993) capability approach.

22 Ten percent of the sample has a safety and security index above 9.5, and five percent is above 9.7. Simply removing the extreme cases in the top five percent yields all results pertaining to safety and security entirely insignificant.

23 We have experimented with using the Human Development Index, which additionally includes education and health, but we do not find that it increases the explanatory value compared to only using GDP per capita (cf. Cahill, 2005). Results are available on request.

24 A note of caution applies to the interaction results, as they may suffer from multiple test bias. Applying a Holm-Bonferoni correction to the standard errors of each of the seven group of estimates leaves the GDP interactions insignificant, while the results for the rule of law in higher-trust and for freedom of assembly in democracies remain stably significant. A conservative reading of the empirical results would thus still leave one with these two main findings.

25 We have also undertaken an interaction analysis between the indicators of legal freedom and income inequality, even though the theoretical link is not as clear as for the other proposed mediators. The results suggest that the rule of law matters for satisfaction with freedom when income inequality is below a certain, quite low level, but we do not obtain significant results for most of the range of values of the Gini coefficient. Results are available on request.

26 One may regard certain kinds of legal freedom as more foundational than others, and we suggest that the one we find to be most strongly related to satisfaction with freedom, freedom of association, assembly and civil society, could fruitfully be regarded as such. If it is in place, it may, in practice, entail other, more specific types of freedom. As a simple indication in support of this, we find that of the countries with a value of freedom of association, assembly and civil society below 5 (on the 10-point scale) in our sample, only 10% have values above 5 for any other indicator of legal freedom.
